# Label-free sub-micrometer 3D imaging of ciprofloxacin in native-state biofilms with cryo-time-of-flight secondary ion mass spectrometry

**DOI:** 10.1007/s00216-022-04496-4

**Published:** 2023-01-10

**Authors:** Anoosheh Akbari, Anzhela Galstyan, Richard E. Peterson, Heinrich F. Arlinghaus, Bonnie J. Tyler

**Affiliations:** 1 Physikalisches Institut and Center for Soft Nanoscience, University of Münster, Wilhelm-Klemm-Straße 10, 48149 Münster, Germany; 2 Department of Chemistry, Center for Nanointegration Duisburg-Essen (CENIDE) and Centre for Water and Environmental Research (ZWU), University of Duisburg-Essen, Universitätsstrasse 5, 45141 Essen, Germany

**Keywords:** ToF–SIMS, Cryogenic analysis, Biofilm, Antibiotic, Multivariate analysis, 3D imaging

## Abstract

High spatial resolution mass spectrometry imaging has been identified as a key technology needed to improve understanding of the chemical components that influence antibiotic resistance within biofilms, which are communities of micro-organisms that grow attached to a surface. Time-of-flight secondary ion mass spectrometry (ToF–SIMS) offers the unique ability for label-free 3D imaging of organic molecules with sub-micrometer spatial resolution and high sensitivity. Several studies of biofilms have been done with the help of ToF–SIMS, but none of those studies have shown 3D imaging of antibiotics in native-state hydrated biofilms with cell-level resolution. Because ToF–SIMS measurements must be performed in a high-vacuum environment, cryogenic preparation and analysis are necessary to preserve the native biofilm structure and antibiotic spatial distribution during ToF–SIMS measurements. In this study, we have investigated the penetration of the antibiotic ciprofloxacin into *Bacillus subtilis* biofilms using sub-micrometer resolution 3D imaging cryo-ToF–SIMS. *B. subtilis* biofilms were exposed to physiologically relevant levels of ciprofloxacin. The treated biofilms were then plunge-frozen in liquid propane and analyzed with ToF–SIMS under cryogenic conditions. Multivariate analysis techniques, including multivariate curve resolution (MCR) and inverse maximum signal factor (iMSF) denoising, were used to aid analysis of the data and facilitate high spatial resolution 3D imaging of the biofilm, providing individually resolved cells and spatially resolved ciprofloxacin intensity at “real world” concentrations.

**Graphical Abstract:**

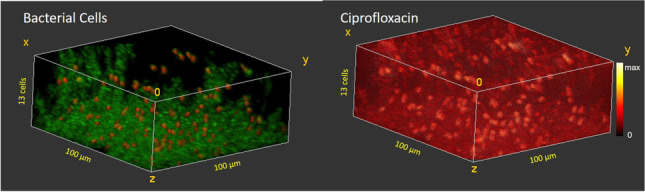

**Supplementary Information:**

The online version contains supplementary material available at 10.1007/s00216-022-04496-4.

## Introduction

Biofilms are communities of microorganisms which grow attached to a surface and are embedded in a self-produced extracellular polymeric substance (EPS) mainly composed of polysaccharides, proteins and extracellular DNA. Microbial biofilms can cause severe health problems such as persistent medical device associated infections and delays in wound healing. They play a role in various diseases such as periodontitis and cystic fibrosis [1]. However, biofilms are also found in the healthy gastrointestinal tract and play an important role in gut homeostasis. Depending on their structure and composition, gut biofilms can provide protection against pathogenic organisms or, alternatively, contribute to disease states [2].

In a biofilm, the viscous EPS matrix can protect bacteria from environmental fluctuations such as pH, temperature, and nutrient concentration and even lead to an antibiotic resistance [3]. Mass transport resistance in the extracellular matrix promotes the formation of heterogeneous micro-environments within the biofilm that vary in the concentration of oxygen, nutrients, waste products, and small molecules such as antibiotics, metabolites, and cell-signaling molecules. Antibiotics have reduced efficacy against biofilm bacteria, as the biofilm bacteria have several mechanisms for combating antibiotics [4]. One of these mechanisms involves the extracellular biofilm matrix acting as a physical barrier to interaction with antibiotics, which inhibits the penetration of antibiotics into the biofilms. The biofilms may also be able to neutralize antibiotics by releasing enzymes or may restrict antibiotic action by depletion of oxygen or pH gradients. Additionally, the biofilm can alter microbial metabolism and gene expression [5]. When the molecular targets of antibiotics are inactive in biofilm embedded cells, this can reduce the effectiveness of antibiotics [3, 4]. In order to better understand the mechanisms of antibiotic resistance in biofilms, improved tools are needed for measuring the 3D distribution of biomolecules, antibiotics, metabolites, and cell-signaling molecules within the biofilm.

Confocal laser scanning microscopy (CLSM) has been widely used to study the 3D distribution of cells, extracellular matrix components, and antibiotics in bacterial biofilms [6, 7]. CLSM is, however, limited to the study of molecules that are either auto-fluorescent or which have been fluorescently tagged. For small molecules, like ciprofloxacin, the fluorescent tag can radically alter transport and binding of the molecule being studied [8].

High spatial resolution mass spectrometry imaging has been identified as a key technology needed to improve the understanding of the chemical language that influences antibiotic resistance within biofilms [9]. Time-of-flight secondary ion mass spectrometry (ToF–SIMS) offers the unique ability for label-free 3D imaging of organic molecules with sub-micrometer spatial resolution and high sensitivity.

In high spatial resolution ToF–SIMS, the surface is bombarded with a highly focused pulsed analytical beam. In state-of-the-art instruments, liquid metal ion guns (LMIG) are used to produce highly focused (< 100 nm) cluster ions such as Bi_3_^+^ or Au_3_^+^. These species are able to desorb intact molecular ions of lipids, peptides, and pharmaceuticals from the outermost 1 to 2 nm of the sample surface. Desorbed ions are then analyzed using a time-of-flight mass analyzer. The beam is rastered across the surface to generate mass spectral images. High repetition rates (5 to 10 kHz) allow for rapid acquisition of images. To generate 3D images, a dual-beam mode is commonly employed. The sample is first imaged using a highly focused analysis beam such as Bi_3_^+^, and then a second high current ion beam (sputter beam) is used to remove a layer of material. By alternating between imaging and sputtering steps, 3D images can be measured with a full mass spectrum at each image voxel. Large Ar gas clusters have become widely used in the sputter step due to their ability to sputter organic materials without leaving residual damage in the sample. Recently, commercial instruments have added MS/MS capability or a dual analyzer option to assist with peak assignment.

The utility of ToF–SIMS for studying freeze-dried bacterial cells has been long established [10–17]. Recently, several studies of biofilms have been done with the help of ToF–SIMS, but none of those studies have shown 3D imaging of antibiotics in native-state hydrated biofilms with cell-level resolution.

ToF–SIMS measurements must be performed in a high vacuum environment (< 10^−8^ mbar) so specialized sample preparation techniques such as freeze-drying or cryogenic analysis are required for the analysis of biological specimens. ToF–SIMS has been previously used to detect quinolones, surfactants, nanoparticles, and antibiotics in biofilms [18–23]; however, the majority of these studies were performed on fixed and dried biofilms leading to artifacts in the 3D structure and potential redistribution of small molecules. Hua et al. have made measurements of hydrated biofilms through a 2-µm orifice in a micro-fluidic chamber but were unable to obtain 3D images of cells and cell clusters because of the small size of the orifice [24–27]. Tyler et al. published 2D images of the anti-microbial chlorhexidine gluconate in cross-sections of frozen hydrated *Candida albicans* biofilms in 2006 [28]. However, this work was done before the widespread availability of cluster ion sources so 3D images were not possible and a high concentration of the anti-microbial was necessary because of the low sensitivity to molecular ions when using Ga^+^ primary ions. More recently, J. Zhang et al. [29] published 3D molecular images of frozen hydrated *Pseudomonas aeruginosa* biofilms using a combined Orbitrap/ToF–SIMS instrument. However, that work was performed using > 1-µm spatial resolution so the cell level structure of the biofilm was not observed and antibiotics were not included in the study.

In this study, we have investigated the penetration of the antibiotic ciprofloxacin into *Bacillus subtilis* biofilms using sub-micrometer resolution 3D imaging cryo-ToF–SIMS. *B. subtilis* is a non-pathogenic gram-positive bacteria found in soil and in the gastrointestinal tract of ruminants and humans. *B. subtilis* has been the major model for the study of gram-positive organisms for many decades and has become an important model system for the study of biofilm formation [9]. In this work, *B. subtilis* biofilms were exposed to physiologically relevant levels of ciprofloxacin. The biofilms were then plunge-frozen in liquid propane and analyzed with ToF–SIMS under cryogenic conditions to preserve the native structure of the biofilm and the spatial distribution of the ciprofloxacin. Multivariate analysis techniques, including multivariate curve resolution (MCR) [30] and inverse maximum signal factor (iMSF) denoising [31], were used to aid analysis of the data and facilitate high spatial resolution 3D images of the biofilm with individually resolved cells and spatially resolved ciprofloxacin over a range of concentrations.

## Experimental

### Biofilm growth

*B. subtilis,* strain DB104 (clinical isolate, University Hospital Münster), was used in this study. The biofilms were grown on specially prepared aluminum planchets that were 4.6 mm in diameter and 620 µm thick with an 80 µm recess. In order to improve attachment of the biofilm to the aluminum surface, the substrates were first coated with thin polydopamine film obtained by pH-induced, oxidative polymerization of dopamine-hydrochloride in alkaline solutions. Polydopamine was applied to the aluminum surfaces by immersing the substrates in a dilute aqueous dopamine solution (2 mg/ml dopamine (Sigma-Aldrich) in 10 mM TRIS buffer (Sigma-Aldrich)) and stirring for 18 h. This wet coating method is suitable for almost all organic and inorganic surfaces. A weakly alkaline condition is a general requirement for oxidative self-polymerization of dopamine. TRIS buffer in our case had a pH of 8.5 [32]. Samples were sterilized by immersion in 70% EtOH for 2 to 3 h and then washed vigorously with sterilized water. For biofilm growth, an overnight culture of the *B. subtilis* was diluted 1:100 into lysogeny broth (LB broth), and 300 μL aliquots were transferred to the wells of a 48-well polystyrene microtiter plate (Nunc®) containing the polydopamine-coated aluminum substrates. The biofilms were then allowed to grow in an incubator at 37 °C for 2 to 4 weeks. The medium was changed every 3 days. Prior to cryofixation, the bacterial biofilm samples were removed from the medium and washed with 150 mM ammonium formate solution (Sigma-Aldrich). The ammonium formate was used as a cryoprotectant and has previously been shown to have minimal interference with ToF–SIMS analysis [29].

The biofilm samples were then exposed for 5 min to solutions of 150 mM ammonium formate containing ciprofloxacin (Sigma-Aldrich) varying in concentration from 1000 to 10 µg/ml. The lowest concentration, 10 µg/ml, is near the minimum inhibitory concentration for ciprofloxacin and is a realistic “real world” level of the antibiotic [20]. The samples were then plunge-frozen in liquid propane. To prevent deposition of a frost layer on the samples, the plunge-freezing procedure was done inside a glovebox containing a dry nitrogen atmosphere. Samples were transferred to a copper sample stub and kept under liquid nitrogen until samples were transferred from the glovebox to the ToF–SIMS instrument. Samples were transferred from the glove box to a pre-cooled sample holder in the ToF–SIMS load-lock using a Bal-Tec VCT 100 shuttle [28, 29, 33] to prevent deposition of water and other contaminants from ambient air onto the frozen sample.

### ToF–SIMS analysis

ToF–SIMS measurements were performed using a custom instrument built by IONTOF GmbH that is largely equivalent to the IONTOF M6. The instrument is equipped with direct liquid-nitrogen closed loop circulation cryo-stages in both the load-lock and the main chamber which allowed for precise (± 2 °C) temperature control throughout the measurements [29]. All measurements were performed in dual beam, non-interlaced mode using a 30 keV Bi_3_^+^ primary ion beam for analysis, and an 18 nA 20 keV Ar_2000_^+^ sputter beam. The sputter area was specified as 500 × 500 $\mu$ m^2^, and the analysis area was 100 × 100 $\mu$ m^2^. A 2.4 A electron beam was used for charge compensation. Measurements were performed in positive polarity mode, because preliminary studies showed very low ion yield for ciprofloxacin in negative ion mode. The temperature was set at − 140 °C in both the main chamber and the load-lock in order to prevent sublimation of water from the frozen sample. At this temperature, no change in the sample was observed over a 24-h period.

Data was collected in two different modes, a high speed low lateral resolution mode and a high lateral resolution mode. For each ciprofloxacin concentration, three depth profiles were measured in the high speed low lateral resolution mode using a bunched 0.2 pA 30 keV Bi_3_^+^ primary ion beam and the “All Purpose” analyzer settings using a 200 µs cycle time. This provided ~ 2 µm lateral resolution and a mass resolution of over 6000 m/Δm. These measurements were made in non-interlaced mode with 2 analysis frames, 1 shot/pixel per frame in each scan with the raster in random mode. Each analysis cycle was followed by 3 sputter frames and a 2 s pause for surface charge stabilization.

For each ciprofloxacin concentration, a single 3D high spatial resolution image was collected using the LMIG fast imaging mode. A 30 keV Bi_3_^+^ un-bunched pulsed beam with a current of 0.05 pA and a measured lateral resolution of better than 200 nm was used. For the high spatial resolution 3D images, ‘’Delayed extraction LMIG’’ analyzer settings were used, with a cycle time of 110 $\mu$s, 5 analysis frames per cycle, non-interlaced mode with 2 sputter frames per cycle followed by a 2 s pause. The analysis frames were done in random raster mode with 512 × 512 pixels over a 100 × 100 µm^2^ analysis area.

## Results and discussion

**Fig. 1:**
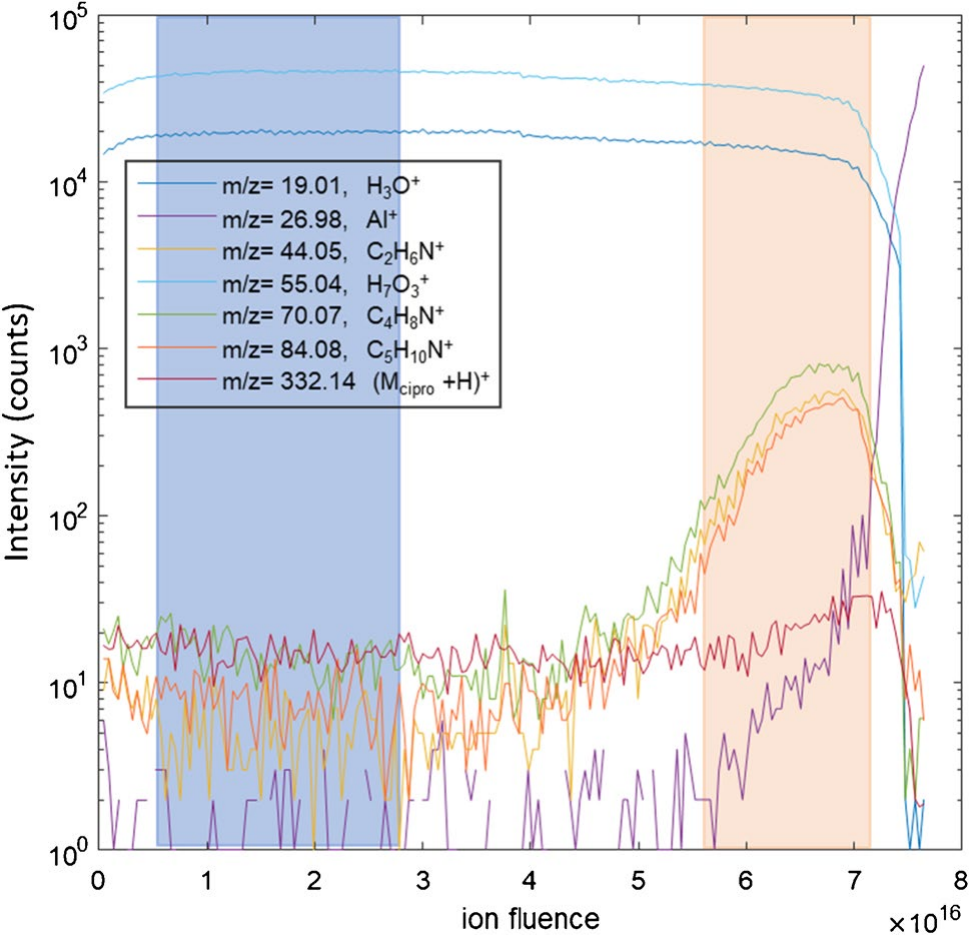
Selected ion signals from ToF–SIMS depth profile through a *B. subtilis* biofilm exposed for 5 min to 50 µg/ml of ciprofloxacin. The cell-free frozen aqueous layer is highlighted in blue, and the biofilm layer is highlighted in orange

For all measurements, the dominant peaks in the mass spectra were water cluster ions, including both peaks of the formula H_2n__+1_O_n_^+^ with smaller peaks at masses corresponding to H_2n_O_2_^+^ ions. No decline in the water ion peaks was observed after more than 24 h in high vacuum. Figure 1 shows selected ion depth profiles for the low lateral resolution analysis of a biofilm sample exposed to 50 µg/ml ciprofloxacin. The depth profile is typical of the measurements and shows three regions: an outer aqueous layer containing only water, ammonium formate and ciprofloxacin which is highlighted in blue, a biofilm layer indicated by peaks at m/z 70.07, m/z 44.05, and 84.08 which is highlighted in orange, and finally the aluminum substrate below the biofilm. The peak at m/z 70.07 has been previously identified as an indicator of gram-positive cells [14], and the peaks at m/z 44.05 and m/z 84.08 are characteristic fragments of alanine and lysine, respectively [34]. Both amino acids are an important constituent of the gram-positive cell wall [11]. The three layers observed in Fig. 1 were found in all of the low lateral resolution depth profiles. Figure 1 also shows the ciprofloxacin (M + H)^+^ signal. Although the depth profiles are shown on a log scale, an increase in the ciprofloxacin (M + H)^+^ signal is observable in the biofilm layer. For each of the low lateral resolution data sets, a region was selected in the outer, biofilm-free, aqueous layer, and a region was selected in the biofilm layer, as illustrated in Fig. 1. For each ciprofloxacin concentration, the ratio of the ciprofloxacin (M + H)^+^ signal to the H_25_O_12_^+^ signal was calculated for the aqueous layer and the biofilm layer. Results are summarized in Table 1.

**Table 1:** Ciprofloxacin (M_cipro_ + H)^+^ to water H_25_O_12_^+^ intensity ratio, low lateral resolution depth profiles

Ciprofloxacin concentration	Aqueous layer intensity ratio	Biofilm layer intensity ratio
10 µg/ml	0.0060 ± 0.0003	0.013 ± 0.003
50 µg/ml	0.012 ± 0.0004	0.022 ± 0.003
100 µg/ml	0.015 ± 0.0007	0.020 ± 0.005
1000 µg/ml	0.11 ± 0.008	0.21 ± 0.05

In the aqueous layer, there is a linear relationship between the (M_cipro_ + H)^+^ to H_25_O_12_^+^ ratio and the ciprofloxacin concentration (*R*^2^ = 0.999). The ratio does not, however, go to zero at zero concentration due to the high background from metastable water cluster decay. In the biofilm layer, in contrast, the relationship between the concentration and the (M_cipro_ + H)^+^ to H_25_O_12_^+^ is not linear in the low concentration range and the variability between measured regions is greater. This may be attributable to variability in the cell density and biomass concentration between different regions of the biofilm.

For each of the 4 ciprofloxacin concentrations and a biofilm without ciprofloxacin, a single 3D ToF–SIMS image was measured. The raw ToF–SIMS data for the five 3D images is available in generic raw data (GRD) format via Zenodo [35]. A peak list was generated by the Surface Lab 7.4 peak search routine, and high spatial resolution 3D images were processed with MCR using Surface Lab 7.4. The MCR analysis facilitated the identification of two cell types along with factors that described variations in the ice spectrum due to detector non-linearity. MCR factor spectra for the two cell types are shown in the supporting material (Fig. S3). A factor describing the ciprofloxacin alone was not observed for any of the samples. To more accurately determine the distribution of the ciprofloxacin in the biofilms, the raw data was exported to MATLAB and processed using iMSF denoising [31]. For the iMSF denoising, 1090 peaks were selected using an automated peak search routine. The same peak list was used for all the images and 10 factors were used in the data reconstruction for all samples. The denoised images were validated using tests previously described [31]. For all samples, there was no lack of fit (*F*_*j*_ < 0.99) for the ciprofloxacin (M + H)^+^ peak (m/z = 332.14). There was also better than 0.99 correlation (*R*_*j*_^*2*^) between the layer-by-layer mean in the denoised m/z 332.14 signal and the layer-by-layer mean in the raw m/z = 332.14 signal. The 3D images were also segmented in MATLAB, using the MCR factors and a means-clustering algorithm, in order to identify 3D volumes containing the two different cell types, cell-free volumes, and substrate volumes of the images. These volumes were used to validate the denoised ciprofloxacin images, and in all cases the denoised mean m/z 332.14 signal in these volumes was within 10% of the raw data mean, and the correlation coefficient (*R*_*j*_^*2*^) was greater than 0.99.

MCR analysis of the high spatial resolution data revealed two different cell types in the biofilm which was not exposed to ciprofloxacin: “live cells” that contained K^+^, Na^+^ and Ca^+^ ions and metabolically “inactive cells” that showed only organic signals. Potassium, sodium, and calcium that were present in the growth media were removed by the ammonium formate wash, so the only remaining ions were those bound to or trapped within the bacteria. The retention of K^+^ in the cells is an indication that the cells were “live”, that is maintaining potassium homeostasis at the time of cryo-fixation and had an intact cell membrane. The average spectra from the two cell types and the surrounding media were obtained from the raw data using the 3D segmentation performed in MATLAB. Spectra from the high spatial resolution biofilm that was not exposed to ciprofloxacin are shown in the supporting material Fig. S2. Water cluster ions dominate in all three spectra, demonstrating effective retention of water throughout the measurement. Multiple organic peaks are also observed in both cell types. Table 2 shows key signals associated with the biofilm. These include fragments associated with glycine, alanine, and lysine, which are major constituents of the *B. subtilis* cell wall.

**Table 2:** Ion signals associated with *B. subtilis* biofilm cells

Biofilm characteristic ions	Probable source	Mass number (m/z)
CH_4_N^+^	Glycine (protein)	30.03
C_2_H_6_N^+^	Alanine (PE lipid head group fragment)	44.05
C_3_H_4_NO^+^	Asparagine	70.05
C_4_H_8_N^+^	Gram-positive bacteria, proline	70.07
C_4_H_6_NO^+^	Glutamic acid	84.04
C_5_H_10_N^+^	Lysine	84.08
C_5_H_12_N^+^	PC lipid headgroup fragment	86.10
C_4_H_5_N_3_OH^+^	Cytosine	112.05
C_5_H_5_N_5_H^+^	Adenine	136.06
C_5_H_5_N_5_OH^+^	Guanine	152.05
C_8_H_16_NO_6_^+^	N-acetyl-glucosamine	222.10
C_11_H_20_NO_8_^+^	N-acetylmuramic acid	294.12

Signals from n-acetyl-glucosamine and n-acetylmuramic acid, which are also important *B. subtilis* cell wall constituents, are also observed. Additionally, signals from cytosine, adenine, and guanine are associated with the cells. Signals from thymine (m/z = 127.05), meso-diaminopimelic acid (m/z = 128.07), and the phosphatidylethanolamine (PE) lipid head group (m/z 142.03) are not observed due to overlap with high intensity water ion cluster signals. Intact lipids in the higher mass range are also not observed due to strong signals from water ion clusters and a high background resulting from metastable decay of the water cluster ions. The same organic signals are observed in both the “live” cells and the “inactive” cells at nearly equal intensity. The major difference observed between these cell types is the presence of K^+^ ions in the “live” cells, an indication that these cells were maintaining K^+^ homeostasis at the time of cryo-fixation. Eukaryotic and prokaryotic cells maintain an elevated concentration of K^+^ inside the cell as a critical part of homeostasis. The presence of potassium inside cells has long been used in ToF–SIMS analysis as an indicator of cell viability at the time of cryo-fixation [36, 37]. Maintenance of potassium homeostasis is well established as essential for bacterial viability and pathogenicity [38]. Additionally, potassium leakage has been associated with formation of the biofilm cell phenotype in *B. subtilis* [39]. Further studies comparing ToF–SIMS imaging with confocal imaging are needed to better understand the two cell types we have designated as “live” and “inactive” and the relationship of these two cell types to various cell phenotypes that have been previously observed in biofilms.

No signals were observed that could be unambiguously attributed to the extra-cellular polysaccharide matrix. This is unsurprising because polysaccharides typically have low ion yield in positive ion ToF–SIMS, and there is a strong overlap between the common polysaccharide fragment ions and the ice clusters.

**Fig. 2:**
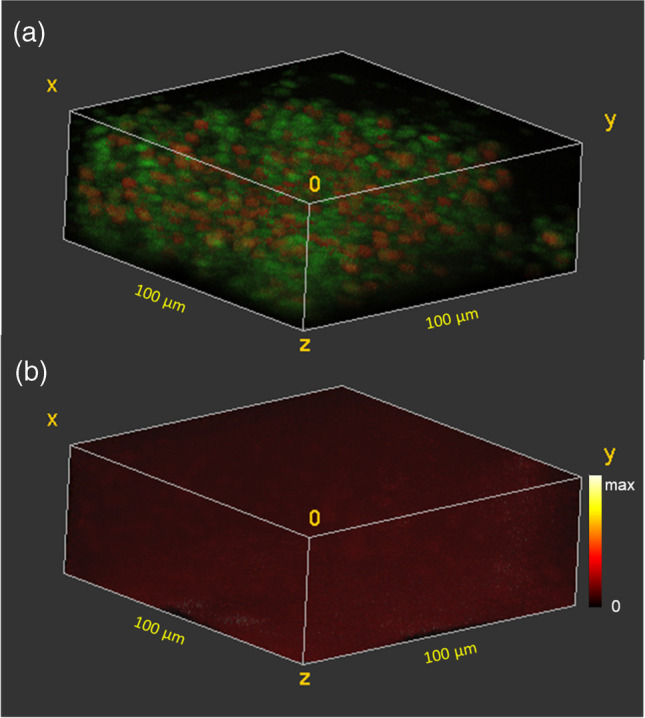
3D ToF–SIMS image of a frozen hydrated *B. subtilis* biofilm prior to exposure to ciprofloxacin. **a** “Live” (red) and “inactive” (green) cells. **b** Background signal at m/z 332.14 where the ciprofloxacin (M + H)^+^ signal would appear

Figure 2a shows the 3D distribution of the two cell types in the biofilm that had no exposure to ciprofloxacin. The “live” cells which contain K^+^ are shown in red and the “inactive” cells in green. The image is 100 µm × 100 µm in the *x* and *y* dimensions, but the *z* scale is uncalibrated due to the unknown sputter yield for ice using 20 keV Ar_2000_^+^ ions. From sections of the 3D image, it was determined that an average of 8 sputter cycles were required to sputter through an individual cell (data not shown), which allows for a rough approximation of the *z* scale in terms of “cell layers.” At the base layer of the untreated biofilm, there are 145 cells in the 100 µm × 100 µm area, of which 53 are live. In the lower eight layers of the biofilm, the cell density remains fairly constant varying between 132 and 166 cells. Above the eighth cell layer, the cell density decreases until only 26 cells are observed in the outermost layer. The percent live cells in the layers varies between the layers from 21 to 51%, with an average of 36%, but no trend is observed with depth. As a control, the iMSF denoised 3D distribution of the background in the region of the ciprofloxacin (M + H)^+^ (m/z 332.14) is shown in Fig. 2b. The denoised background is evenly distributed in 3D with no significant change either with depth or where cells are present.

**Fig. 3:**
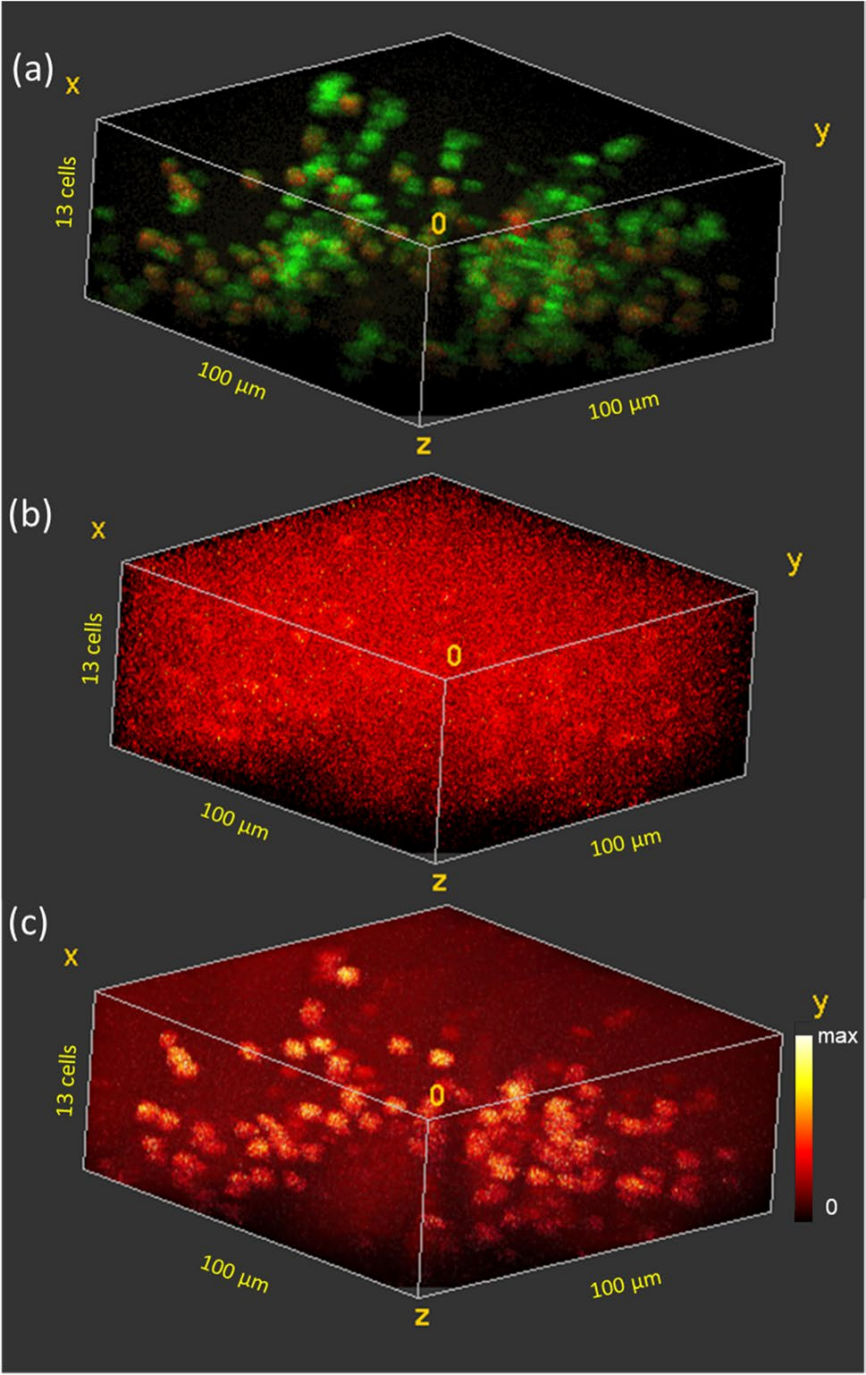
3D ToF–SIMS image of a frozen hydrated *B. subtilis* biofilm after 5 min exposure to 1000 µg/ml ciprofloxacin. **a** “Live” (red) and “inactive” (green) cells. **b** Raw ciprofloxacin (M + H)^+^ signal (m/z 332.14) (8 voxel binning). **c** iMSF denoised ciprofloxacin (M + H)^+^ signal showing preferential binding of ciprofloxacin to the “live” cells

Figure 3a shows the 3D distribution of the two cell types in the biofilm treated with 1000 µg/ml ciprofloxacin. The same two “live” and “inactive” cell types seen in the untreated biofilm are observed with similar spectral signatures to the untreated cells. Live cells are shown in red and inactive cells in green. The cell density in this sample is far lower than in the untreated biofilm. In the deepest 6 cell layers of the biofilm, the cell density varies between 39 and 54 cells in the 100 µm × 100 µm area. The cell density then decreases in the outer 7 cell layers until only one cell is observed in the outermost layer. Live cells constitute 45% of the cells in the sampled volume. The live cells are randomly distributed through the biofilm showing no trend with depth or with regard to cell clusters. Figure 3b shows the raw 3D ciprofloxacin (M + H)^+^ signal. This signal has been down-binned by 2 × 2 × 2 voxels. Non-uniformity in ciprofloxacin signal is evident with cell size volumes showing higher ciprofloxacin signal. Figure 3c shows the iMSF denoised ciprofloxacin (M + H)^+^ signal. Ciprofloxacin is clearly higher intensity in the cells than in the surrounding media, and the highest intensity is observed in the live cells, indicating that the antibiotic is not only penetrating to the base of the biofilm, it is also binding to the cells. Significantly lower cell density was observed in this sample than in the untreated biofilm or in any of the samples exposed to lower concentrations of ciprofloxacin, suggesting that the very high concentration of antibiotic may have disrupted the biofilm. Nonetheless, nearly half the cells observed appear to be “live”, i.e., maintaining potassium homeostasis. Ciprofloxacin inhibits bacterial cell replication rather than disrupting the cell membrane so it is not surprising for the cells to bind ciprofloxacin without releasing intracellular potassium. Ciprofloxicin acts by binding to bacterial DNA topoisomerase and DNA-gyrase [40], so it is also reasonable that it would have higher affinity for living cells than for inactive cells.

**Fig. 4:**
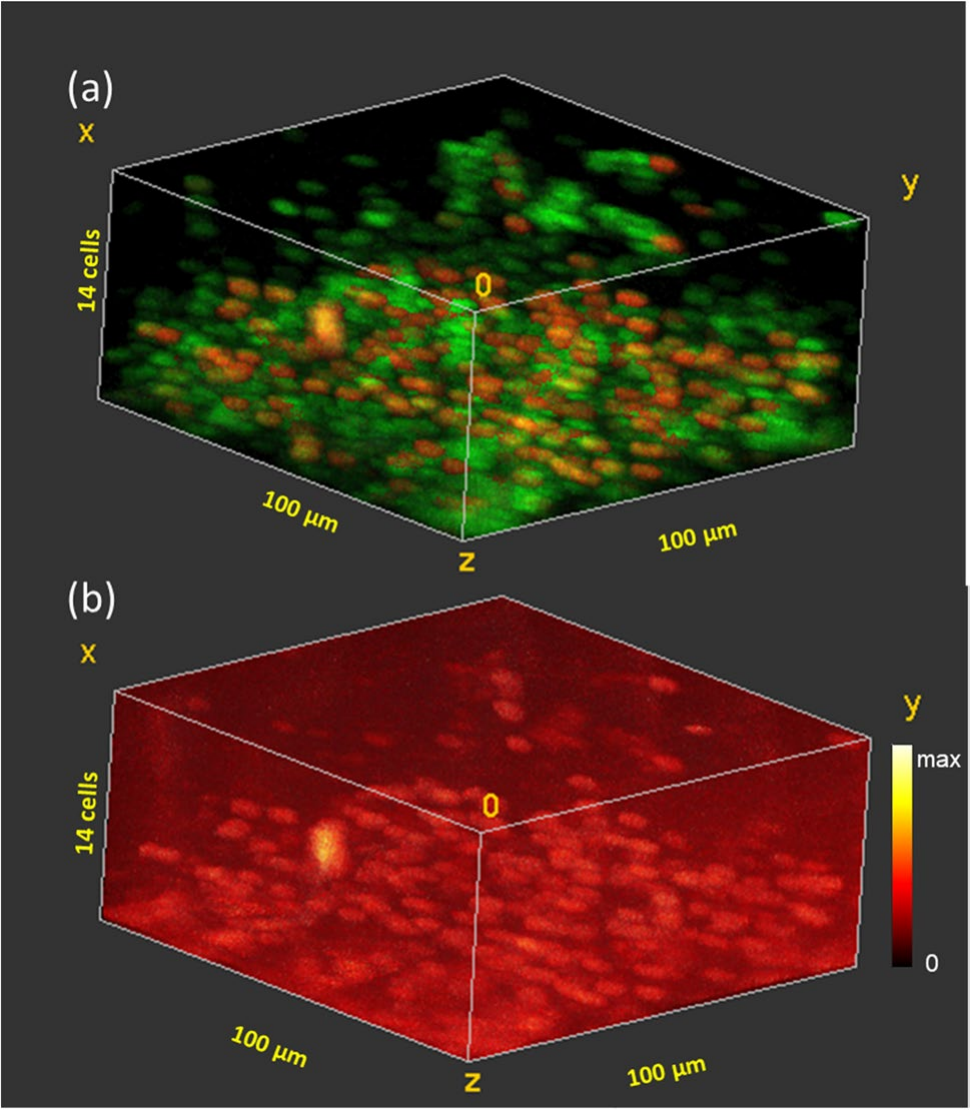
3D ToF–SIMS image of a frozen hydrated *B. subtilis* biofilm after 5 min exposure to 100 µg/ml ciprofloxacin. **a** “Live” (red) and “inactive” (green) cells. **b** iMSF denoised ciprofloxacin (M + H)^+^ signal showing preferential binding of ciprofloxacin to the “live” cells

**Fig. 5:**
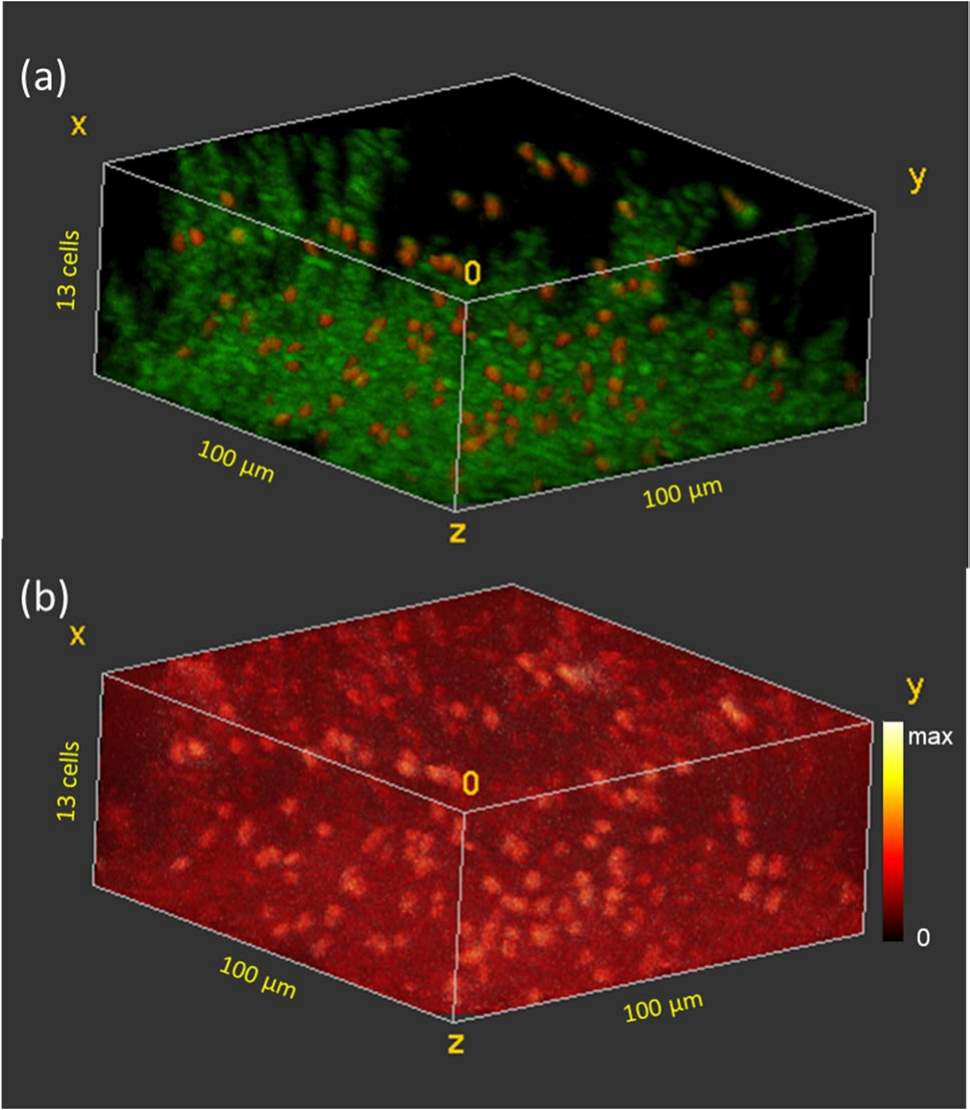
3D ToF–SIMS image of a frozen hydrated *B. subtilis* biofilm after 5 min exposure to 50 µg/ml ciprofloxacin. **a** “Live” (red) and “inactive” (green) cells. **b** iMSF denoised ciprofloxacin (M + H)^+^ signal showing preferential binding of ciprofloxacin to the “live” cells

**Fig. 6:**
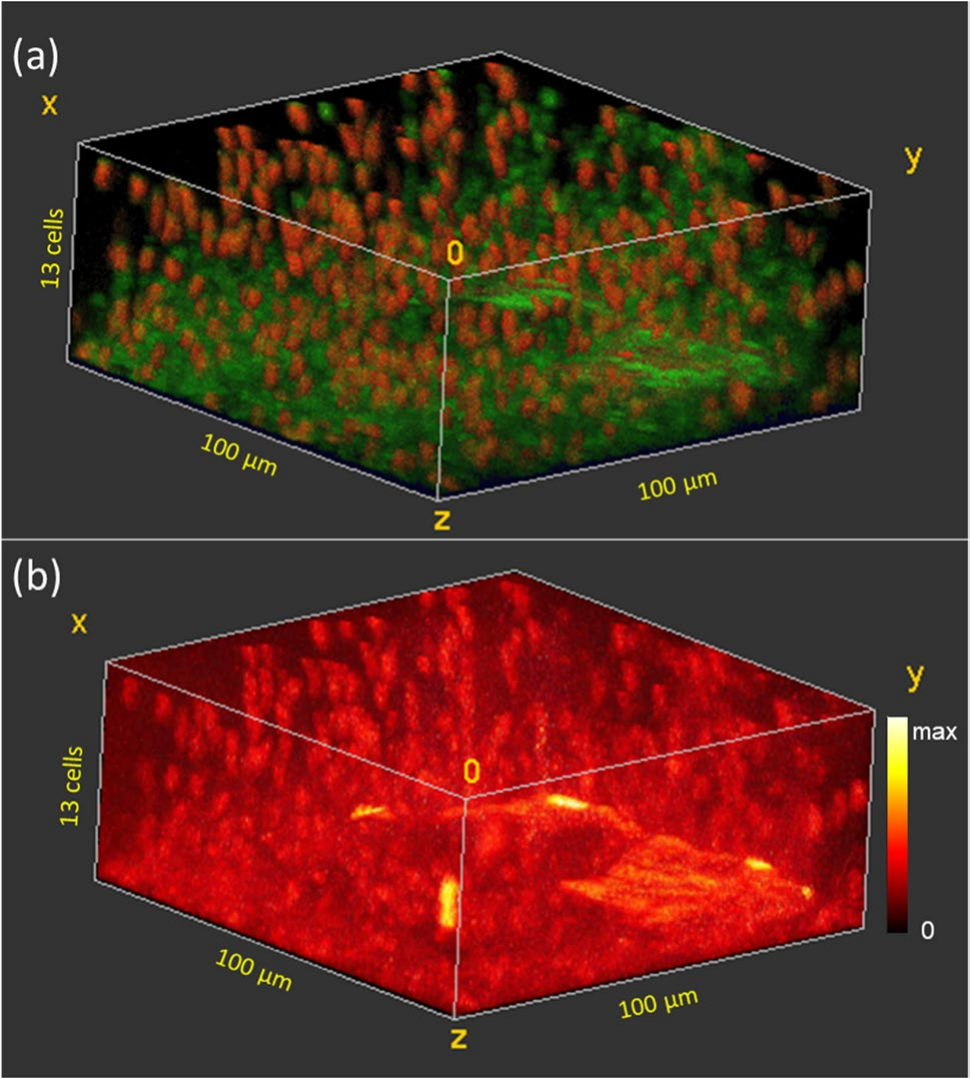
3D ToF–SIMS image of a frozen hydrated *B. subtilis* biofilm after 5-min exposure to 10 µg/ml ciprofloxacin. **a** “Live” (red) and “inactive” (green) cells. **b** iMSF denoised ciprofloxacin (M + H)^+^ signal showing preferential binding of ciprofloxacin to the “live” cells

Figures 4, 5 and 6 show the 3D distribution of cells and ciprofloxacin (M + H)^+^ in the biofilms treated with 100 µg/ml, 50 µg/ml, and 10 µg/ml of ciprofloxacin, respectively. In Figs. 4a, 5a and 6a, the “live” cells are shown in red and the “inactive” cells in green. In Figs. 4b, 5b and 6b, the 3D distribution of the iMSF denoised ciprofloxacin (M + H)^+^ signal is shown. The sample treated with 100 µg/ml ciprofloxacin (Fig. 4a) shows a similar cell distribution to that observed in the untreated biofilm. In the lower 6 cell layers, the number of cells varies between 93 and 123 after which the number of cells declines until only 19 cells are observed in the uppermost layer. The 3D image reveals several columns of cells extending into the upper layers. Forty-one percent of the cells observed are live, and the live cells are once again randomly distributed through the biofilm. Although the raw ciprofloxacin (M + H)^+^ signal intensity was too low to observe patterns in the distribution (data not shown), the iMSF denoised (M + H)^+^ clearly shows increased signal intensity associated with the live cells and a less pronounced increased intensity in the inactive cells relative to media.

The biofilm treated with 50 µg/ml ciprofloxacin (Fig. 5a) also shows a dense cell structure in the bottom layers of the biofilm and then several thin pillars of cells extending into the upper layers. The cells in this sample and in the sample treated with 10 µg/ml ciprofloxacin appear smaller than in the previous images. These biofilm samples were allowed to grow for 4 weeks, and the differences in cell size are attributed to the age of the biofilm. The base biofilm layer contains 382 cells, and the number of cells decreases in each layer approaching the surface of the biofilm. In this sample, only 15% of the cells were live, and there is a trend toward a lower percentage of live cells near the base of the biofilm. As with the other treated biofilms, the ciprofloxacin (M + H)^+^ signal is most intense in the live cells (Fig. 5b).

The biofilm treated with 10 µg/ml (Fig. 6a) also shows a dense cell structure in the bottom layers of the biofilm with between 229 and 280 cells/layer in the bottom 4 layers. After this the number of cells per layer decreases steadily, reaching a level of 77 cells in the outermost layer. In this sample, 30% of the cells are live, and the percent live cells decreases from 66% at the outer surface to only 13% at the base of the biofilm. As with the other treated biofilms, the ciprofloxacin (M + H)^+^ signal is most intense in the live cells (Fig. 6b).

In all four samples treated with antibiotic, the ciprofloxacin signal is strongly correlated with the location of the “live” cells. Close inspection also reveals an elevated ciprofloxacin signal in the “inactive” cells as compared to the cell-free volumes. To further validate this finding, the mean intensity of the ciprofloxacin (M + H)^+^ signal in the “live “ cells, “inactive” cells, and cell-free aqueous volumes was calculated from the raw data without multivariate denoising. In all cases, the ciprofloxacin signal from the combined cell types was found to be significantly greater than the ciprofloxacin signal from the cell-free volumes (*p* < 0.0001, using a Fisher’s exact test). Similarly, the signal from the “live” cells was found to be significantly greater than the signal from the “inactive” cells (*p* < 0.0001, using a Fisher’s exact test).

Table 3 shows the mean (M + H)^+^/H_37_O_18_^+^ ratio for the “live” cells, “inactive” cells, and the cell-free aqueous volumes. For all samples, the ciprofloxacin signal intensities in the cell-free volumes are close to the expected value while the ciprofloxacin signal intensities in both the “live” cells and the “inactive” cells is significantly elevated. The ciprofloxacin intensity in the “live” cells is roughly double the intensity in the cell-free volumes at all ciprofloxacin levels. While matrix effects are always possible in mass spectrometry, suppression of the (M + H)^+^ ion in favor of the (M + K)^+^ ion is the predominant matrix effect that has been observed due to the presence of K^+^. Since we observe an enhancement of the ciprofloxacin (M + H)^+^ ion rather than a suppression, the difference is almost certainly due to a higher concentration of ciprofloxacin rather than a matrix effect. The data indicate that ciprofloxacin penetrates to the base of the biofilm after a 5-min exposure and that ciprofloxacin binds to the cell biomass at a level higher than is observed in the cell-free aqueous surroundings. Furthermore, “live” cells bind the ciprofloxacin more effectively than the “inactive” cells that do not contain potassium.

**Table 3:** High-resolution 3D images: normalized ciprofloxacin intensity

Ciprofloxacin concentration µg/ml	Ratio of [M + H]^+^ to H_37_O_18_^+^			
	Inactive cells	Live cells	All cells	Media
**1000**	3.3E-01	6.3E-01	4.0E-01	2.2E-01
**100**	2.1E-02	3.1E-02	2.3E-02	1.3E-02
**50**	1.4E-02	3.0E-02	1.4E-02	6.9E-03
**10**	1.1E-02	1.9E-02	1.2E-02	2.9E-03
**0**	1.9E-03	3.2E-03	2.0E-03	7.4E-04

## Conclusions

This work demonstrates that cryo-ToF–SIMS analysis can be used to obtain 3D images of ciprofloxacin in native-state biofilms with cell-level resolution. The structures observed in the *B. subtilis* biofilms, such as channels and pillars, are similar to those that have been previously observed using confocal microscopy imaging. With the aid of multivariate analysis techniques, we were able to identify and localize “live” cells, which were maintaining potassium homeostasis, and “inactive” cells, which did not contain potassium. We were able to measure the 3D distribution of ciprofloxacin at “real world” concentrations with the aid of iMSF denoising. In this study, after an exposure time of 5 min, ciprofloxacin was not only detected through the full thickness of the biofilm; it was found preferentially bound to the cell biomass and particularly to the “live” cells. These findings were only made possible through the combination of sub-µm LMIG primary ion analysis, argon cluster ion sputtering, cryo-analysis, and advanced multivariate data analysis. The study demonstrates that high lateral resolution cryo-ToF–SIMS is a powerful tool for the study of antibiotics in biofilms. The ability to measure antibiotics with cell-level resolution in the native biofilm state and without the need for labels that can alter the behavior of the antibiotic is critical for elucidation of the mechanisms associated with antibiotic resistance in biofilms. Further study combining cryo-ToF–SIMS with complementary techniques such as confocal microscopy, Orbitrap-SIMS, and MALDI-MSI promises to advance understanding of antibiotic resistance in biofilms.

## Supplementary Information

Below is the link to the electronic supplementary material.

Supplementary file1 (DOCX 270 kb)

## References

[1] Muhsin J, Ufaq T, Tahir H, Saadia A (2015). Bacterial biofilm: its composition, formation and role in human infections. J Microbiol Biotechnol.

[2] Motta J-P, Wallace JL, Buret AG, Deraison C, Vergnolle N (2021). Gastrointestinal biofilms in health and disease. Nat Rev Gastroenterol Hepatol.

[3] Castrillón-Rivera Laura E, Palma-Ramos A, Marina P (2012). Biofilms: a survival and resistance mechanism of microorganisms. Antibiotic Resistant Bacteria.

[4] Stewart PS, William CJ (2001). Antibiotic resistance of bacteria in biofilms. The Lancet.

[5] Khot PD, Suci PA, Miller RL, Nelson RD, Tyler BJ (2006). A small subpopulation of blastospores in Candida albicans biofilms exhibit resistance to amphotericin B associated with differential regulation of ergosterol and beta-1,6-glucan pathway genes. Antimicrob Agents Ch.

[6] Reichhardt C, Parsek MR (2019). Confocal laser scanning microscopy for analysis of pseudomonas aeruginosa biofilm architecture and matrix localization. Front Microbiol..

[7] Palmer RJ, Sternberg C (1999). Modern microscopy in biofilm research: confocal microscopy and other approaches. Curr Opin Biotech.

[8] Dollery CT (2013). Intracellular Drug Concentrations. Clin Pharmacol Ther.

[9] Arnaouteli S, Bamford NC, Stanley-Wall NR, Kovács ÁT (2021). Bacillus subtilis biofilm formation and social interactions. Nat Rev Microbiol.

[10] Tian H, Six DA, Krucker T, Leeds JA, Winograd N (2017). Subcellular chemical imaging of antibiotics in single bacteria using C60-secondary ion mass spectrometry. Anal Chem.

[11] Tyler BJ (1997). XPS and SIMS studies of surfaces important in biofilm formation. Three case studies. Ann N Y Acad Sci..

[12] Thompson CE, Jungnickel H, Lockyer NP, Stephens GM, Vickerman JC (2004). ToF-SIMS studies as a tool to discriminate between spores and vegetative cells of bacteria. Appl Surf Sci.

[13] Tuccitto N, Marletta G, Carnazza S, Grasso L, Caratozzolo M, Guglielmino S (2011). ToF-SIMS imaging of surface self-organized fractal patterns of bacteria. Surf Interface Anal.

[14] Vaidyanathan S, Fletcher JS, Jarvis RM, Henderson A, Lockyer NP, Goodacre R (2009). Explanatory multivariate analysis of ToF-SIMS spectra for the discrimination of bacterial isolates. Analyst.

[15] Al-Bataineh SA, Jasieniak M, Britcher LG, Griesser HJ (2008). TOF-SIMS and principal component analysis characterization of the multilayer surface grafting of small molecules: antibacterial furanones. Anal Chem.

[16] Wehrli PM, Lindberg E, Angerer TB, Wold AE, Gottfries J, Fletcher JS (2014). Maximising the potential for bacterial phenotyping using time-of-flight secondary ion mass spectrometry with multivariate analysis and tandem mass spectrometry. Surf Interface Anal.

[17] Nilsson KD, Palm M, Hood J, Sheriff J, Farewell A, Fletcher JS (2019). Chemical changes on, and through, the bacterial envelope in Escherichia coli mutants exhibiting impaired plasmid transfer identified using time-of-flight secondary ion mass spectrometry. Anal Chem.

[18] Osorio JHM, Benettoni P, Schmidt M, Stryhanyuk H, Schmitt-Jansen M, Pinto G (2019). Investigation of architecture development and phosphate distribution in Chlorella biofilm by complementary microscopy techniques. Fems Microbiol Ecol..

[19] Benettoni P, Stryhanyuk H, Wagner S, Kollmer F, Osorio JHM, Schmidt M (2019). Identification of nanoparticles and their localization in algal biofilm by 3D-imaging secondary ion mass spectrometry. J Anal Atom Spectrom.

[20] Davies SK, Fearn S, Allsopp LP, Harrison F, Ware E, Diggle SP (2017). Visualizing antimicrobials in bacterial biofilms: three-dimensional biochemical imaging using TOF-SIMS. mSphere..

[21] Lanni EJ, Masyuko RN, Driscoll CM, Aerts JT, Shrout JD, Bohn PW (2014). MALDI-guided SIMS: multiscale imaging of metabolites in bacterial biofilms. Anal Chem.

[22] Seyeux A, Zanna S, Allion A, Marcus P (2015). The fate of the protective oxide film on stainless steel upon early stage growth of a biofilm. Corros Sci.

[23] Wei W, Zhang Y, Komorek R, Plymale A, Yu R, Wang B (2017). Characterization of syntrophic Geobacter communities using ToF-SIMS. Biointerphases.

[24] Hua X, Marshall MJ, Xiong YJ, Ma X, Zhou YF, Tucker AE (2015). Two-dimensional and three-dimensional dynamic imaging of live biofilms in a microchannel by time-of-flight secondary ion mass spectrometry. Biomicrofluidics..

[25] Hua X, Yu XY, Wang ZY, Yang L, Liu BW, Zhu ZH (2014). In situ molecular imaging of a hydrated biofilm in a microfluidic reactor by ToF-SIMS. Analyst.

[26] Ding Y, Zhou Y, Yao J, Szymanski C, Fredrickson J, Shi L (2016). In situ molecular imaging of the biofilm and its matrix. Anal Chem.

[27] Ding Y, Zhou Y, Yao J, Xiong Y, Zhu Z, Yu X-Y (2019). Molecular evidence of a toxic effect on a biofilm and its matrix. Analyst.

[28] Tyler BJ, Rangaranjan S, Möller J, Beumer A, Arlinghaus HE (2006). TOF-SIMS imaging of chlorhexidine-digluconate transport in frozen hydrated biofilms of the fungus Candida albicans. Appl Surf Sci.

[29] Zhang J, Brown J, Scurr DJ, Bullen A, MacLellan-Gibson K, Williams P (2020). Cryo-OrbiSIMS for 3D molecular imaging of a bacterial biofilm in its native state. Anal Chem.

[30] Tyler BJ, Rayal G, Castner DG (2007). Multivariate analysis strategies for processing ToF-SIMS images of biomaterials. Biomaterials.

[31] Tyler BJ, Kassenböhmer R, Peterson RE, Nguyen DT, Freitag M, Glorius F (2022). Denoising of mass spectrometry images via inverse maximum signal factors analysis. Anal Chem..

[32] Lee H, Dellatore SM, Miller WM, Messersmith PB (2007). Mussel-inspired surface chemistry for multifunctional coatings. Science.

[33] Möller J, Beumer A, Lipinsky D, Arlinghaus HF (2006). Introduction of a cryosectioning-ToF-SIMS instrument for analysis of non-dehydrated biological samples. Appl Surf Sci.

[34] Wagner MS, Castner DG (2001). Characterization of adsorbed protein films by time-of-flight secondary ion mass spectrometry with principal component analysis. Langmuir.

[35] Akbari A, Galstyan A, Peterson RE, Arlinghaus HF, Tyler BJ. Cryo-ToF-SIMS 3D images of ciprofloxacin in Bacillus subtilis biofilms. Zenodo. 2022.

[36] Malm J, Giannaras D, Riehle MO, Gadegaard N, Sjövall P (2009). Fixation and drying protocols for the preparation of cell samples for time-of-flight secondary ion mass spectrometry analysis. Anal Chem.

[37] Fartmann M, Dambach S, Kriegeskotte C, Lipinsky D, Wiesmann HP, Wittig A (2003). Subcellular imaging of freeze-fractured cell cultures by TOF-SIMS and laser-SNMS. Appl Surf Sci.

[38] Do EA, Gries CM (2021). Beyond homeostasis: potassium and pathogenesis during bacterial infections. Infect Immun.

[39] López D, Fischbach MA, Chu F, Losick R, Kolter R (2009). Structurally diverse natural products that cause potassium leakage trigger multicellularity in Bacillus subtilis. Proc Natl Acad Sci U S A.

[40] Thai TS, BH; Zito PM ciprofloxacin: StatPearls; 2022 [Available from: https://www.ncbi.nlm.nih.gov/books/NBK535454/. Accessed 29 Jul 2022.

