# Supplementary material for: Label-free sub-micrometer 3D imaging of ciprofloxacin in native-state biofilms with cryo-time-of-flight secondary ion mass spectrometry
Source: Anal Bioanal Chem. 2023 Jan 10;415(5):991–9. doi: 10.1007/s00216-022-04496-4 (PMC9883301; doi:10.1007/s00216-022-04496-4)
Supplement: Supplementary file 1 — Supplementary file1 (DOCX 270 kb) [file 216_2022_4496_MOESM1_ESM.docx]

**Supplemental Material**

**Label-free Sub-Micrometer 3D Imaging of Ciprofloxacin in Native-State Biofilms with Cryo-Time-of-Flight Secondary-Ion-Mass Spectrometry**

Anoosheh Akbari^1^, Anzhela Galstyan^2^, Richard E. Peterson^1^, Heinrich F. Arlinghaus^1^, and Bonnie J. Tyler^1*^

1. Physikalisches Institut and Center for Soft Nanoscience, University of Münster, Wilhelm-Klemm-Straße 10, Münster, Germany
2. Department of Chemistry, Center for Nanointegration Duisburg-Essen (CENIDE) and Centre for Water and Environmental Research (ZWU), University of Duisburg-Essen, Universitätsstrasse 5, 45141 Essen.

* Corresponding author email: [tyler@uni-muenster.de](mailto:tyler@uni-muenster.de)

**Table of Contents.**

Figure S1

Figure S2

Figure S3


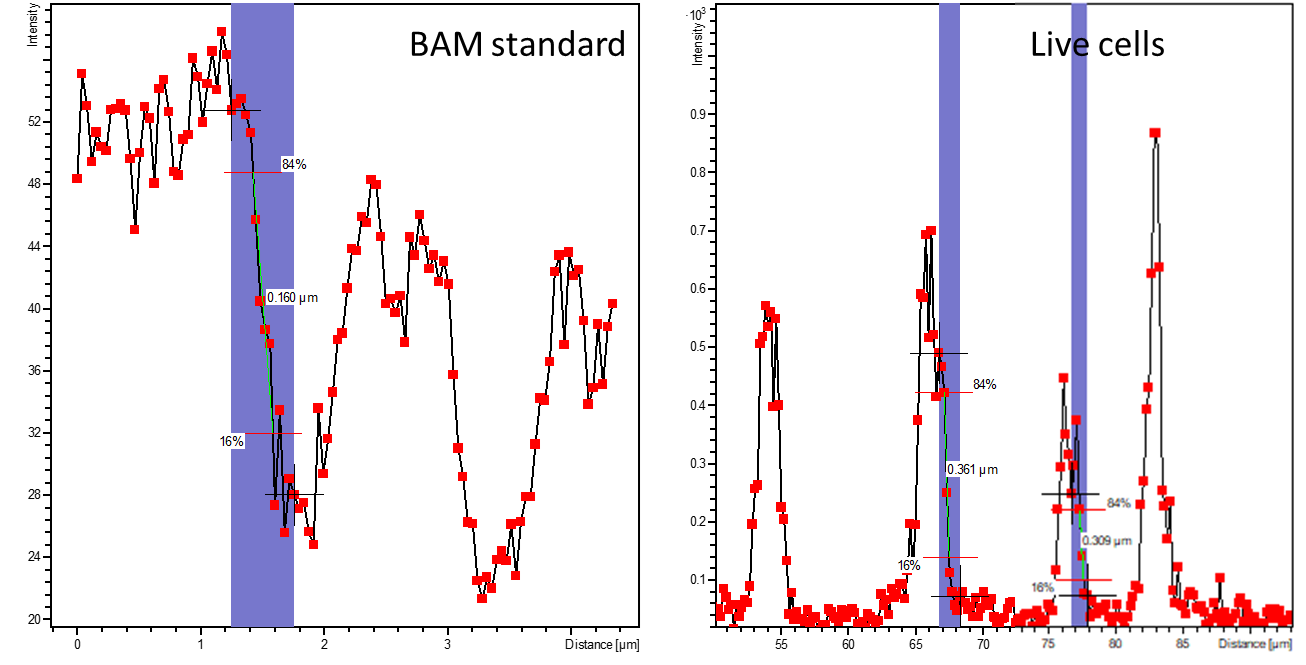


Figure S1: The lateral resolution from a line scan on resolution standard BAM-L200 is less than 200 nm. A line scan across the MCR image of the live cells shows a measured edge resolution in the 300 nm range. Because the edge of the cell is not a sharp edge, the measured resolution will be larger.


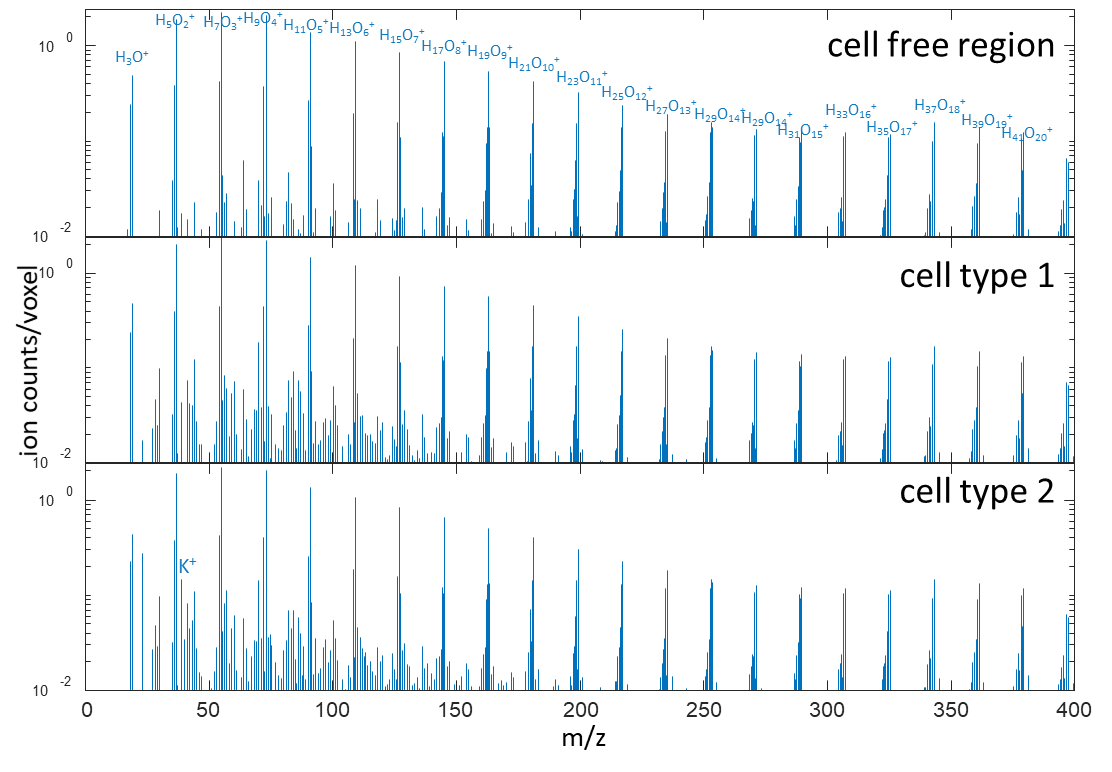


Figure S2: Cryo-ToF-SIMS spectra from three volumes in a *B. subtilis* biofilm that had not been treated with antibiotic. The dominant peaks in the cell-free volume, cell type 1 and cell type 2 are from water clusters. The volumes of interest were generated in 3 dimensions in MatLab.


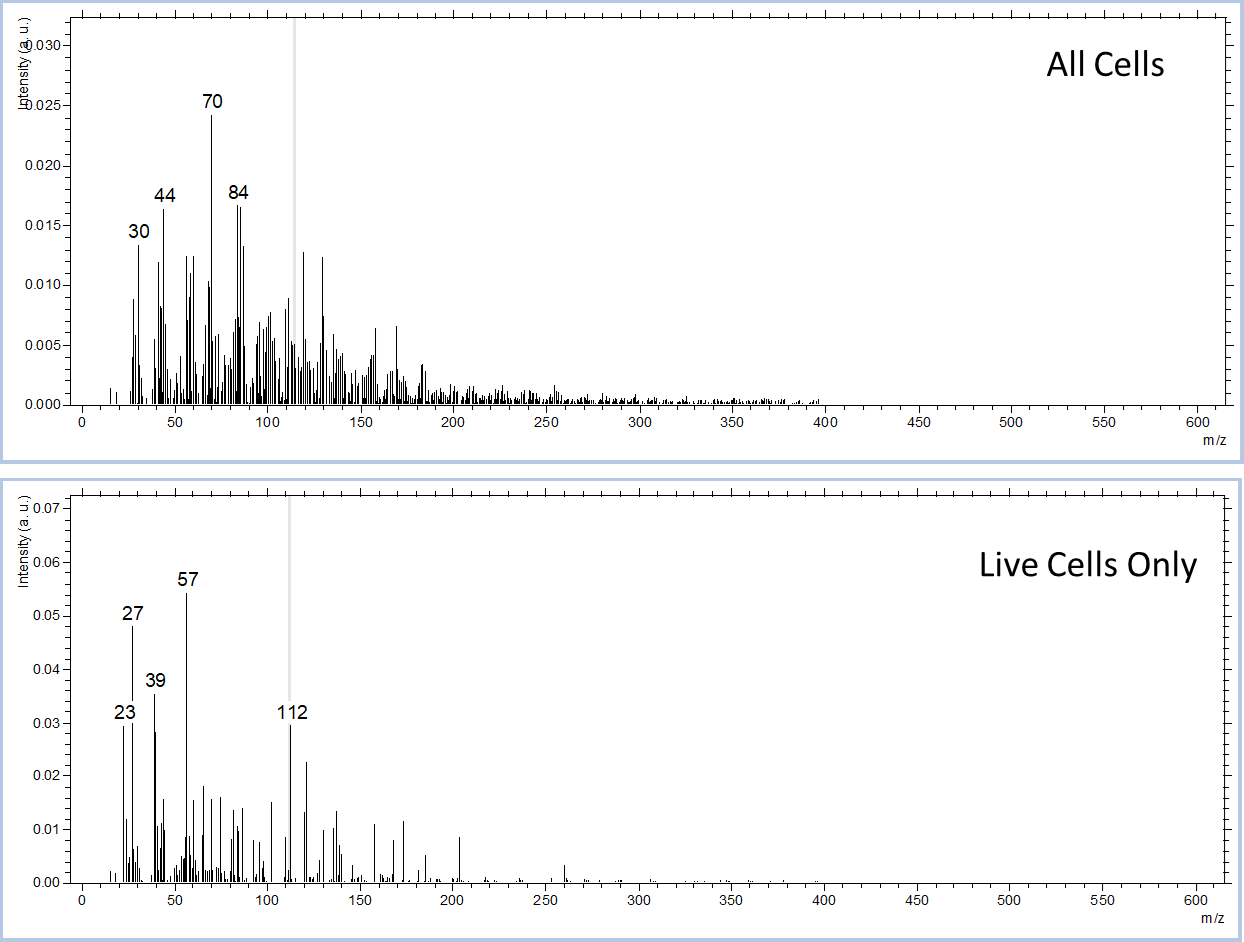


Figure S3: MCR factor spectra for all of the cells and for only the live cells.
